# Outcomes of a hypertension care program based on task-sharing with private pharmacies: a retrospective study from two blocks in rural India

**DOI:** 10.1038/s41371-023-00837-7

**Published:** 2023-05-19

**Authors:** Hemanshu Das, Ashish Sachdeva, Harish Kumar, Ashish Krishna, Andrew E. Moran, Anupam K. Pathni, Bhawna Sharma, Bhanu P. Singh, Manish Ranjan, Sarang Deo

**Affiliations:** 1 Indian School of Business, Hyderabad, India; 2 Yale School of Management, Yale University, New Haven, CT USA; 3 Resolve to Save Lives, New Delhi, India; 4 Resolve to Save Lives, New York, NY USA; 5 Division of General Medicine, Columbia University Irving Medical Center, New York, NY USA; 6 Nanocare Health Services, Hyderabad, India

**Keywords:** Preventive medicine, Diagnosis

## Abstract

Low density of formal care providers in rural India results in restricted and delayed access to standardized management of hypertension. Task-sharing with pharmacies, typically the first point of contact for rural populations, can bridge the gap in access to formal care and improve health outcomes. In this study, we implemented a hypertension care program involving task-sharing with twenty private pharmacies between November 2020 and April 2021 in two blocks of Bihar, India. Pharmacists conducted free hypertension screening, and a trained physician offered free consultations at the pharmacy. We calculated the number of subjects screened, initiated on treatment (enrolled) and the change in blood pressure using the data collected through the program application. Of the 3403 subjects screened at pharmacies, 1415 either reported having a history of hypertension or had elevated blood pressure during screening. Of these, 371 (26.22%) were enrolled in the program. Of these, 129 (34.8%) made at least one follow-up visit. For these subjects, the adjusted average difference in systolic and diastolic blood pressure between the screening and follow-up visits was −11.53 (−16.95 to −6.11, 95% CI) and −4.68 (−8.53 to −0.82, 95% CI) mmHg, respectively. The adjusted odds of blood pressure being under control in this group during follow-up visits compared to screening visit was 7.07 (1.29 to 12.85, 95% CI). Task-sharing with private pharmacies can lead to early detection and improved control of blood pressure in a resource-constrained setting. Additional strategies to increase patient screening and retention rates are needed to ensure sustained health benefits.

## Background

About 55 million Indians are estimated to suffer from cardiovascular diseases (CVDs) causing an economic loss of $94 billion annually. India accounts for one-fifth of the global deaths due to CVDs [1]. India’s health and economic burden from CVD is expected to increase further due to an aging and growing population [2–4]. High blood pressure (or hypertension) is the most important preventable risk factor for CVDs and accounts for more than half of the CVD deaths [5]. Over the past twenty years, the prevalence of uncontrolled hypertension in rural India has increased faster than in urban areas owing, in part, to rapid urbanization, lack of formal care in the private sector, and weak non-communicable disease programs in the public sector [6].

Rural India is estimated to have one allopathic doctor (formal care) for every 10,000 individuals [7–9] (compared to WHO recommendation of 1:1000) leading many patients to seek care very late or from informal care providers [10]. In such resource-limited settings, task-sharing, the practice of transferring some of the appropriate primary duties from the physician to allied healthcare professionals (e.g., nurses, pharmacists, community health workers), has been found to be effective for managing a range of health conditions [11]. However, although half of India’s 800,000 pharmacies are located in rural areas and usually act as the first point of healthcare access [8, 12], evidence on the feasibility and effectiveness of involving them in task-sharing programs for managing hypertension is limited [13].

In this study, the Bihar Pharmacist Hypertension Study, we trained community pharmacists in rural India to assist in hypertension screening, diagnoses, treatment, and follow-up without any direct monetary incentives for the pharmacists. We studied the effect of this task-sharing intervention on the care provided to hypertensive individuals and the resulting health outcomes.

## Methods

### Study design

The Bihar Pharmacist Hypertension Study was a retrospective cohort study to evaluate the effect of a task-sharing program with community pharmacists on the control and reduction of blood pressure in resource-constrained rural Indian settings.

### Study setting

The program was rolled out in two blocks (a primary administrative division of a district) in the Bhojpur district of Bihar (India), Behea and Udwantnagar (Supplementary Fig. 1). The two blocks have a population and area of approximately 300,000 and 350 sq km respectively [14], with an estimated adult hypertension prevalence of 24% [15]. We mapped 65 licensed pharmacies in the two blocks and selected 20 pharmacies based on their location (proximity to town centers and arterial roads), the strength of the cellular signal received, availability of space to conduct screenings and consultations, self-reported patient volume (prioritizing higher volume pharmacies), and pharmacists’ willingness to participate. The program was implemented from 23rd November 2020 to 30th April 2021. We excluded individuals below 18 years old and pregnant women from the study. We excluded the blocks wherein the state health administrators were running a separate hypertension initiative through the primary health centers.

### Study intervention

Each pharmacy received a validated digital blood pressure monitor and a hand-held tablet device with a pre-installed application that stored patients’ electronic health records. The pharmacists were trained on using the application and performing the activities in the program through a full-day training session. Study field staff regularly visited the pharmacists to assist with difficulties in the program’s day-to-day activities. The program adopted the standardized hypertension treatment protocol followed under the India Hypertension Control Initiative (IHCI) [16]. The pharmacies were recommended to stock the generic, low-cost, antihypertensive medication (amlodipine, telmisartan, and chlorthalidone) based on the hypertension treatment protocol adopted under the IHCI program [16].

**Fig. 1:**
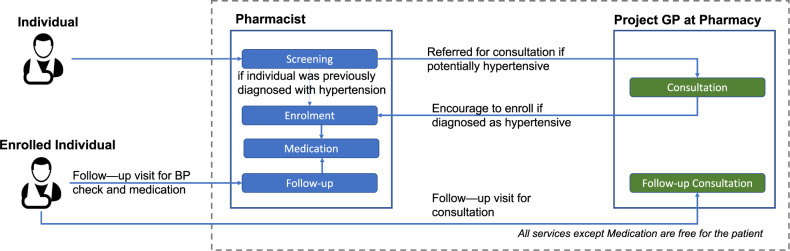
The arrows show an individual’s movement through the various steps in the Bihar Pharmacist Hypertension Study. It starts with a blood pressure screening by the pharmacist, and then the individual follows the flow diagram.

Figure 1 illustrates the patient flow in the study.

### Screening

Pharmacists screened adults visiting the pharmacy by measuring their blood pressure for free and stored the patients’ electronic health records in the program application after taking informed consent in the local language. Subjects with a self-reported history of hypertension were classified as *previously diagnosed*. Subjects with no known history of hypertension were classified, based on their blood pressure reading, as either *normotensive* (systolic blood pressure (SBP) < 140 mmHg and diastolic blood pressure (DBP) < 90 mmHg) or *previously undiagnosed* (SBP ≥ 140 mmHg or DBP ≥ 90 mmHg). The *previously undiagnosed* and *previously diagnosed* groups were collectively classified as *potentially hypertensive*.

### Consultations

A trained allopathic physician (GP) was recruited for the program duration. The GP visited each pharmacy based on a predetermined schedule at monthly intervals and offered free consultations to all individuals identified as potentially hypertensive on the first screening. The pharmacists informed potentially hypertensive subjects about the future visit date(s) of the GP at the pharmacy. Using the tablet device, the pharmacist could book a consultation for the subject with the project GP on a specific date and time. To ensure equitable access and incentivize screening, normotensive subjects were also provided free GP consultation but did not have the option to pre-book an appointment. During the consultation, the GP confirmed the diagnosis of the potentially hypertensive subjects and prescribed medication based on the standardized hypertension treatment protocol [16]. The pharmacists dispensed the medication as per the GP prescription. The medication expenses were out-of-pocket expenditure for the patients.

### Enrollments and follow-ups

Subjects with confirmed diagnosis of hypertension (previously diagnosed and undiagnosed) were encouraged to enroll in the program for free follow-ups with the pharmacy and the GP. Previously diagnosed individuals had the option to enroll in the program without the project GP diagnosis as well. Enrolled subjects were encouraged to follow-up with the pharmacy monthly and record their blood pressure readings and refill medication if required. Additionally, subjects received follow-up reminder calls from the pharmacists to promote medication adherence and follow-up consultations during subsequent scheduled GP visits. At subsequent GP visits, patients received regular hypertension care.

### Data collection

We used the programmatic data collected through the pre-installed application for our analysis. The demographic variables (age, sex) and the self-reported diagnosis status of the subjects were collected at the first visit. The longitudinal data, recorded at every visit, included blood pressure measurements at each visit, an identifier to mark if each visit was a GP consultation or a pharmacy visit, and enrollment status of the subject. As part of the data cleaning, we identified records with missing blood pressure values. If an additional reading was available for the same calendar day and it was valid, that reading was used to replace the missing value. An individual could have multiple readings on the same day if the data was recorded by both pharmacist and doctor, or if either recorded the data twice. We removed records wherein either the SBP/DBP was not recorded or could not be restored, or a 0 value was recorded. We also removed records with unusually high values of SBP (>250 mmHg).

### Blood pressure measurement

All participating pharmacists were provided with an Omron HBP-1320 sphygmomanometer [17]. Pharmacists used a handheld tablet with a custom application developed by Nanocare Health Services to record blood pressure measurements of patients. The application stored demographic information, blood pressure readings, and allowed for scheduling consultations between the pharmacists and GP. The GP was able to record the patient’s diagnosis and hypertensive status, and the data was provided to the researchers in an anonymous form.

The pharmacists were trained on the blood pressure measurement protocol based on the guidelines followed by the IHCI [18]. Blood pressure was measured using a proper size cuff placed on the uncovered upper arm. If the first reading was ≥140 mm Hg or ≥90 mm Hg for SBP or DBP respectively, a second measurement was taken and recorded as the final reading. If the difference in both readings was more than 10 mm Hg, a third blood pressure measurement was taken and recorded as the final reading. The verbatim training module pertaining to the blood pressure measurement is provided in the Supplementary Appendix.

### Analysis

We defined four operational outcomes of the program: (i) the number of subjects screened for hypertension during the program, (ii) the number of subjects who had at least one GP consultation, (iii) the number of subjects enrolled in the program, and (iv) the number of subjects who did a subsequent follow-up visit with either the pharmacist or the GP. The first outcome was disaggregated into three mutually exclusive categories based on their blood pressure at the time of screening and diagnosis history: previously diagnosed with hypertension, previously undiagnosed (no previous history of hypertension and had elevated blood pressure at screening), and normotensive (no previous history of hypertension and had normal blood pressure at screening).

To analyze downstream health outcomes, we considered enrolled subjects with one or more follow-up visits. For each of them, we estimated the change in SBP and DBP and the odds of blood pressure being under control (SBP < 140 mmHg and DBP < 90 mmHg) during follow-up visit(s) compared to the screening visit. To estimate the change, we developed a linear regression model with the dependent variable as the SBP (or DBP) during each visit of the eligible subject. To estimate the odds of blood pressure being under control, we developed a logistic regression model with the binary dependent variable indicating if the subject’s blood pressure was uncontrolled (=0) or controlled (=1). The main explanatory variable of interest for these regression models was a categorical variable indicating if the observation was for the initial visit (=0) or a follow-up visit (=1). We estimated these models without (unadjusted) and with (adjusted) patient-fixed effects. The fixed effects controlled for observed (age, sex, previous diagnosis status, pharmacy) and unobserved fixed patient characteristics. In all models, we clustered the standard errors at the level of a pharmacy. We fitted additional models to assess if the effect of the follow-up visits differed by age, sex, and prior diagnosis of hypertension. The mathematical specifications of these regression models are available in the Supplementary Text.

The operational outcomes were calculated using Python 3.8, and the regression models for the health outcomes were estimated using R 4.0.

## Results

### Operational outcomes

**Fig. 2:**
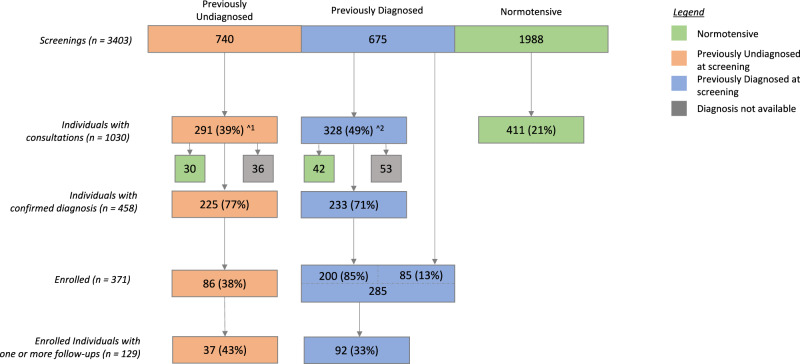
Subject cascade in the program. The numbers in parenthesis indicate the percentage conversion from the previous step. ^^1^27 Previously Undiagnosed individuals had their first consultations post enrolment. ^^2^85 Previously Diagnosed individuals had their first consultations post enrolment.

The baseline characteristics of the subjects screened in the program is tabulated in Table 1. Further, we illustrate the flow of patients through screening, doctor consultation, enrollment if diagnosed as hypertensive and subsequent follows up in Fig. 2.

**Table 1:** Baseline characteristics of subjects in the program.

Baseline characteristics	Screened (*n* = 3403)		Potentially hypertensive (*n* = 1415)		Previously Undiagnosed (*n* = 740)		Previously Diagnosed (*n* = 675)	
Sex n (%)								
Females	1313	(39)	632	(45)	271	(37)	360	(53)
Males	2090	(61)	783	(55)	469	(63)	315	(47)
Age mean (sd)	47	(16)	54	(14)	53	(15)	47	(17)
Systolic BP mean (sd)	134	(23)	152	(24)	155	(20)	133	(22)
Diastolic BP mean (sd)	81	(12)	88	(13)	90	(12)	81	(12)
BP classification *n* (%)								
Controlled^#^			274	(19)	39*	(5)	235	(35)
Uncontrolled			1141	(81)	701	(95)	440	(65)

The unit for systolic and diastolic blood pressure is mmHg; *SD* Standard Deviation.

*39 Previously undiagnosed individuals with BP below 140/90 mmHg were diagnosed with hypertension upon GP consultation.

#Controlled BP: SBP < 140 mmHg and DBP < 90 mmHg.

Uncontrolled BP: SBP > = 140 mmHg or DBP > = 90 mmHg.

### Screening

The program screened 3403 subjects for hypertension. Table 1 shows that 2090 (61%) of the screened subjects were males, and 1313 (39%) were females. The mean (SD) age of the screened subjects was 47.41 (16.01) years. At the initial visit, the mean (SD) SBP and DBP were observed as 133.9 (23.37) mmHg and 80.9 (12.03) mmHg, respectively.

Of the 3403 individuals screened, 1415 subjects were potentially hypertensive. These included 740 (52.3%) previously undiagnosed, and 675 (47.7%) previously diagnosed. Of the 675 previously diagnosed subjects, 440 (65%) had high blood pressure at the time of screening.

### Consultation

Among the 1415 potentially hypertensive subjects at screening, 619 (44%) subjects consulted with the GP. These included 291 (39%) of the 740 previously undiagnosed subjects and 328 (49%) of the 675 previously diagnosed subjects. This difference between the two groups was statistically significant (*p*-value < 0.001). Of the 291 previously undiagnosed subjects who consulted with the GP, 225 (77%) were confirmed with a diagnosis of hypertension. Among the previously diagnosed subjects, 233 (71%) of the subjects were confirmed with diagnosis of hypertension out of the 328 patients who sought consultation.

### Enrollments and follow-ups

Of the 225 previously undiagnosed individuals with confirmed diagnosis, 86 (38%) enrolled in the program and 37 (43%) of these had at least one subsequent follow-up visit. Of the 233 previously diagnosed subjects with confirmed diagnosis from the project GP, 200 (85%) enrolled in the program. An additional 85 previously diagnosed subjects enrolled directly in the program without a GP consultation. Of these 285 previously diagnosed and enrolled subjects, 92 (33%) made at least one subsequent follow-up visit during the project period. There was no statistically significant difference (*p*-value of 0.089) in proportion of patients with at least one follow-up between previously undiagnosed and previously diagnosed groups of patients.

### Downstream clinical outcomes

To evaluate the clinical outcomes, we considered subjects with at least one follow-up visit (*n* = 129) after excluding one subject due to potentially incorrect blood pressure readings at follow-up. Baseline characteristics of this sub-sample of patients are in Supplementary Table 1 whereas their comparison with those of the screened and potentially hypertensive subjects is in Supplementary Table S2. Enrolled subjects with at least one follow-up visit were older and had higher SBP/DBP than the screened subjects and the potentially hypertensive screened subjects but there was no statistically significant difference in the sex composition of these groups.

Among enrolled individuals, assuming patients without a follow-up reading have uncontrolled blood pressure (a pessimistic scenario), the proportion of individuals with controlled BP reduced from 23.72% at time of screening to 11.05% at the end of the program. If we assume that patients without a follow-up reading maintain their blood pressure until end of the program, the proportion of individuals with controlled BP increase to 28.03% at the end of the program.

**Fig. 3:**
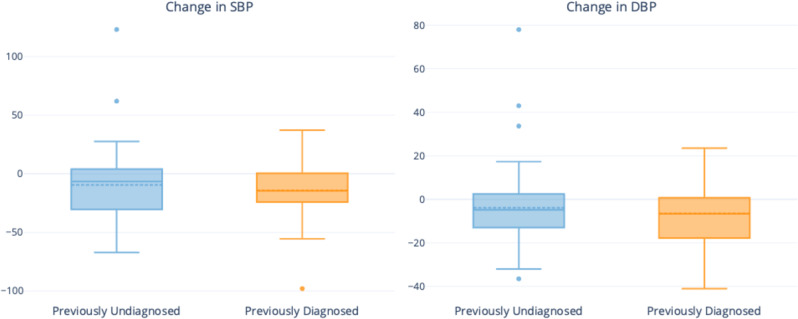
Change in systolic and diastolic blood pressure for enrolled individuals with one or more follow-up visits. Change in SBP (or DBP) (in mmHg) is the difference in SBP (or DBP) at screening and SBP (or DBP) at the last follow-up visit (with negative values indicating a reduction in blood pressure). The box plot represents the Interquartile range with the solid line and dashed line within the box indicating the median and mean. The whiskers indicate the values within ranging between 1.5*IQR below the lower quartile and 1.5*IQR above the upper quartile. The outlier dots are beyond the 1.5*IQR values.

Figure 3 illustrates the difference in the SBP/DBP between the screening visit and the last follow-up visit of the eligible subjects (*n* = 129). The proportion of subjects with controlled blood pressure increased from 13.5% at screening to 35.1% at the last follow-up for previously undiagnosed group and from 21.7% at screening to 30.4% at the last follow-up for previously diagnosed group. Taken together, the proportion rose from 19.2% at screening to 32.3% at the last follow-up among all individuals with one or more follow-up visits. (The artifact that previously undiagnosed individuals were observed with controlled BP occurred since the patients were identified with hypertension during the GP consultation).

The regression results for change in blood pressure with follow-up are tabulated in Table 2. Compared to the initial screening visit, the unadjusted changes in SBP and DBP were −11.52 (−16.63 to −6.4, 95% CI) and −5.02 (−8.23 to −1.82, 95% CI) mmHg, respectively. When adjusted for patient fixed effects, the changes in SBP and DBP were −11.53 (−16.95 to −6.11, 95% CI) and −4.68 (−8.53 to −0.82, 95% CI) mmHg. The unadjusted odds of having controlled blood pressure during follow-up visit(s) compared to screening visit was 2.53 (1.28 to 3.78, 95% CI). When adjusted for subject-fixed effects, the odds of having controlled blood pressure during follow-up visits compared to screening was 7.07 (1.29 to 12.85, 95% CI) upon follow-up.

**Table 2:** Change in systolic and diastolic blood pressure and odds of controlled blood pressure with follow-up among enrolled subjects with one or more follow-up visits (*N* = 129*).

Parameter	Unadjusted (*n* = 129)	Adjusted^#^ (*n* = 129)
Change in Systolic BP (in mmHg)	−11.52 (−16.63 to −6.4, 95% CI)	−11.53 (−16.95 to −6.11, 95% CI)
Change in Diastolic BP (in mmHg)	−5.02 (−8.23 to −1.82, 95% CI)	−4.68 (−8.53 to −0.82, 95% CI)
Odds for BP control during follow-up(s) compared to screening	2.53 (1.28 to 3.78, 95% CI)	7.07 (1.29 to 12.85, 95% CI)

^#^Adjusted for age, sex, prior diagnosis with hypertension, pharmacy, and other unobserved fixed effects.

*One subject was removed from analysis due to erroneous blood pressure reading at follow-up.

We did not observe any statistically significant differences in the outcomes (reduction of SBP/DBP, odds of blood pressure control) between previously undiagnosed and previously diagnosed subjects or between males and females. Older subjects had a greater reduction in SBP with follow-up but no statistical difference in change in DBP or odds of blood pressure control. The detailed regression results are provided in Supplementary Tables S3–S5.

## Discussion

India’s rural population has restricted access to standardized hypertension care due to a severe shortage of formal care providers. Task-sharing is seen as possible means to bridge the gap. The Bihar Pharmacist Hypertension Study provides real-world evidence on the impact of task-sharing with pharmacists for hypertension screening and management. In particular, we find that such task-sharing leads to reduction in SBP and DBP and increased odds of controlling blood pressure for subjects enrolled in the program, who completed at least one follow up visit. However, it is noteworthy that only 35% of the enrolled hypertensive subjects followed for one or more visits over five months of the program. However, among those patients who did follow up, the proportion with controlled blood pressure at last follow up increased by over 12% after five months of the program.

Our findings agree with those from previous studies on task-sharing of hypertension care with pharmacists. A meta-analysis of interventions in LMICs found that team-based hypertension treatment including task-sharing with pharmacists resulted in a change of –8 ∙ 12 (–10 ∙ 23 to –6 ∙ 01, 95% CI) mmHg in SBP and a change of −3.74 (−5.15 to −2.32, 95% CI) mmHg in DBP. A change of −5.8 (−8.2 to −3.2, 95% CI) mmHg in SBP and −7.1 (−9.8 to −4.2) mmHg in DBP was observed among 77 subjects (over six months) receiving pharmacy-led care in a diabetes clinic in Jordan [13, 19]. A randomized controlled trial comprising 129 subjects accessing routine care in China observed a change of −8.5 ( ± 16.45) and −4.7 ( ± 9.76) mmHg for SBP and DBP, respectively [20]. In Thailand, a change of −23.29 ( ± 19.10) and −14.18 ( ± 11.20) mmHg was observed in SBP and DBP for 118 subjects [21]. In all three studies, the patients were aware of their hypertensive status prior to the start of the study and were recruited during their regular visit to the formal care facility for care. Our study extends the existing work by estimating outcomes where subjects might not be aware of their hypertensive status or did not have access to formal care. Further, in all three studies mentioned above, the pharmacists advised and changed the treatment plan based on the patient’s progress in addition to regular monitoring of blood pressure and advice on lifestyle changes. Also, the pharmacists were employed at the health care facility wherein the patients were accustomed to visiting. However, in our study, the pharmacists delivered the intervention in community pharmacy settings separate from formal health care facilities and were limited to measuring blood pressure and scheduling physician visits. Limited pharmacist scope of hypertension care practice was due to lack of the formal training required for providing medical advice among pharmacists in India and lack of government regulations allowing for increased scope of pharmacist practice. Our program also utilized private sector pharmacists who were neither employed by nor received any direct monetary incentives or compensation from the program. A couple of likely unobserved benefits of the program were (i) increased status-awareness of hypertension among subjects who thus might have sought care outside the program, and (ii) improved treatment adherence among previously diagnosed subjects due to the availability of formal care in the community.

Despite the promising impact on blood pressure, the long-term health outcomes from such a program rely on continued adherence to medication and follow-up with the care provider [22, 23]. Only 26% of the screened potentially hypertensive subjects enrolled in the program, and only 35% of them followed up with subsequent visit(s). Although our study duration was only five months, the observed retention was lower than that in previous task-sharing studies from LMICs [24–26]. In a study in rural Cameroon, 62% of the enrolled subjects had subsequent follow-up visits [24]. However, the study involved task-sharing with nurses employed at the clinic. In a task-sharing intervention in the city of Lagos (Nigeria), 51% of the subjects followed up post-enrollment [25]. The study design was similar to ours with the exception of patient participation fee of $0.96/month and the pharmacists receiving a fee for each patient monitored. The barbershop trials in the US reported retention of 95% over a period of six months [27], but participants were provided $25 per pharmacy visit [27].

However, due to the chronic nature of hypertension, financial incentives for treatment adherence might be unsustainable. A possible alternative to increasing patient retention may be creation of community support groups as observed in an intervention conducted in the slums of Nairobi, Kenya [26]. Also, word-of-mouth spread of promising results within the community could increase uptake and retention but validating this hypothesis required further research. Further, the introduction of care coordinators with the specific responsibility of following up with individuals identified as potentially hypertensive could improve both diagnosis rates and health outcomes. A program in the urban slums of Mumbai (India) showed promising results on treatment adherence and blood pressure control by employing care coordinators to assist providers in following up with the hypertensive patients [28].

Patient or pharmacist financial incentives may have improved follow-up in the Bihar Pharmacist Hypertension Study. Our program did not cover patient out-of-pocket expenditure for medication, travel, or wage loss (if any) during pharmacy or GP visits. It is likely that if the program covered such expenses, it would have improved patient retention. A modeling study conducted in India demonstrated that improving patient retention would have significant long-term health and economic benefits [29]. Specifically, a program covering 40% of the population and retaining 40% of diagnosed patients (similar to the performance of the Bihar Pharmacist Hypertension Study) would be cost-effective, resulting in a 0.35% increase in averted disability-adjusted life years and a $3.88 per capita reduction in tertiary care costs. Moreover, the study showed that improving retention could make preventive care programs cost-saving since the reduction in tertiary care costs would exceed the investment in preventive care [29]. Overall, these findings underscore the importance of patient retention as a key factor in achieving positive health outcomes and reducing overall healthcare costs.

Any future scale-up of the program critically hinges on the sustained involvement of the pharmacists delivering the intervention, which also likely depends on the financial benefits accrued to them. The program did not remunerate the pharmacies based on the assumption that pharmacies would benefit from increased pharmaceutical sales due to the intervention and GP visits. However, it is possible that the pharmacies may not perceive the same benefit. Further, it is plausible that the pharmacy catchment area might have limited customers, and the pharmacies might run out of new customers after some time, which may diminish their interest in the program in the long term.

The Bihar Pharmacist Hypertension Study was limited by not including a comparator group who were randomized to follow up in a traditional medical setting after screening. However, a comparator group was not included in the study design due to resource constraints. Geographic coverage was limited to two blocks in a single district of India; these results may not represent the feasibility of a community pharmacy model of hypertension care in other areas of India. However, the study’s setting was representative of large swathes of rural India suffering from poor public health infrastructure and lack of access to formal, standardized care. Our study design did not include data collection on out-of-pocket expenses of patients, and we did not model long-term health benefits. While a scale-up is likely to be beneficial, a future study on the cost-effectiveness analysis of a scaled-up task-sharing program can help compare the approach to other preventive programs. The participation in the program might have also been impacted as the study period partially overlapped with the second wave of the COVID19 pandemic in India (March–April 2021) [30]. The reduced mobility during this period may have caused a reduction in screenings and poor retention in care.

The experiences and outcomes of the Bihar Pharmacist Hypertension Study provide policymakers with a plausible model for collaborating with the private sector to increase access to healthcare for chronic conditions like hypertension in areas with weak public healthcare delivery capacity. Although our program employed its own itinerant GP, other intervention designs could be tested. For example, the GP could supervise pharmacist care by way of telemedicine consultations, or pharmacists could screen patients but refer them to the nearest primary health center in the public health system for diagnosis and follow up care. Similar approach has yielded encouraging results in treating drug-resistant TB in India and identifying new TB cases [31]. Community pharmacy-based hypertension management programs will require increased expenditure but an effective linkage program for hypertension management can avoid developing new infrastructure and trained health worker capacity needed to expand hypertension treatment coverage. Investment cost is also offset by avoided tertiary care spend for treatment of CVDs [29], which has rapidly emerged as one of the largest items of expenditure under state and central government health insurance schemes [32]. Together, these practical economic considerations add up to a strong case for public sector investment in task-sharing preventive care programs that can promise desirable health outcomes for hypertension and other chronic diseases.

## Conclusion

The Bihar Pharmacist Hypertension study demonstrated the feasibility of team-based hypertension care involving community pharmacists in rural India. Hypertension outcomes were favorable among the one-third of enrolled patients who returned for follow up. However, to be successful, team-based care programs involving pharmacists must improve patient retention by focusing on aligning provider and patient incentives to improve quality in terms of patient screenings, enrollment, and retention.

## Summary

### What is known on this topic?

- Task-sharing with non-physician healthcare workers can aid in the management of hypertension in resource-constrained settings.
- Rural India, while lacking formal care facilities, is catered by more than 800,000 private pharmacies.
- There are no known studies of task-sharing with private-sector pharmacies to aid the management of hypertension.

### What this study adds

- Task-sharing with private sector pharmacies can lead to the early detection of hypertension, and lead to improved outcomes in the control of blood pressure in rural India.
- Managing patient retention and incentives for pharmacist participation is important for the success of a task-sharing initiative.

### Supplementary information

Supplementary Appendix

## Data Availability

De-identified data are available on reasonable request. Data reported in this study were a part of the operational data of the implementing organization, Nanocare Health Services. Authors did not have any special privileges to access this data. Other scholars can access the same data for research purposes directly from the implementing organization or by requesting authors.
