# Supplementary material for: Outcomes of a hypertension care program based on task-sharing with private pharmacies: a retrospective study from two blocks in rural India
Source: J Hum Hypertens. 2023 May 19;37(11):1033–9. doi: 10.1038/s41371-023-00837-7 (PMC10632126; doi:10.1038/s41371-023-00837-7)
Supplement: Supplementary file 1 — Supplementary Appendix [file 41371_2023_837_MOESM1_ESM.docx]

# Supplementary Appendix

**Supplementary Text: Blood Pressure Measurement**

The steps in the training module provided to the pharmacists have been listed below verbatim:

Step 1:

- Patients should be seated for at least 5 minutes after arrival. They should not have done any strenuous activity for the last 15 minutes
- Ask the patient to be relaxed

Step 2:

- In the seated position, the patient’s arm should be slightly flexed (slightly folded at the elbow)
- A solid support is to be placed below the patient's arm making the armrest on the table surface

Step 3:

- The elbow should be at the level of the heart or at the level of the chest
- The cuff should be wrapped around the upper arm. It is important to choose proper cuff size and a validated device

Step 4:

- Avoid placing the cuff over clothing. Pull the clothing of the patient higher
- The lower edge of the cuff should be about one inch above the fold at the elbow
- The pipe from the cuff should be in the lower direction
- Instruct the patient to not talk during the Blood pressure measurement

Step 5:

- Legs of the patients should not be crossed during this time.
- Press the “On” button on the digital blood pressure apparatus
- The cuff inflates automatically and then deflates slowly.

Step 6:

- Legs of the patients should not be crossed during this time.
- Press the “On” button on the digital blood pressure apparatus
- The cuff inflates automatically and then deflates slowly.

Step 7:

- The display shows the blood pressure and pulse readings after the readings have stabilized
- Enter the Systolic and the Diastolic blood pressure on the mobile application and follow instructions

**Supplementary Text: Regression Models**

We used regression analysis to estimate the impact of the program on health outcomes. We created a panel dataset from the programmatic data and estimated the change in SBP and DBP and the odds of blood pressure under control (SBP <=140 and DBP <= 90) among enrolled subjects with at least one follow-up visit.

The unadjusted effect in SBP was estimated through the following model:

$${sbp}_{svp} = \beta_{0}+ \beta_{1}{followup}_{svp} + \varepsilon_{svp}$$

where

sbp_svp_ is the systolic blood pressure (in mmHg) of subject *s* at visit *v* and at pharmacy *p*

followup_svp_ is a dummy variable. = 0 for the first visit, and = 1 for follow-up visits*.*

$\varepsilon_{svp}$ is the error term. Standard errors are clustered at the pharmacy level.

The variable of interest is followup_svp_. A significant negative coefficient value would indicate that the SBP reduced during follow-up visits.

The unadjusted effect in DBP was estimated using a similar model:

$${dbp}_{svp} = \beta_{0}+ \beta_{1}{followup}_{svp} + \varepsilon_{svp}$$

where

dbp_svp_ is the diastolic blood pressure (in mmHg) of subject *s* at visit *v* and at pharmacy *p*

followup_svp_ is a dummy variable. = 0 for the first visit, and = 1 for follow-up visits*.*

$\varepsilon_{svp}$ is the error term. Standard errors are clustered at the pharmacy level.

To estimate the unadjusted odds of blood pressure under control, we developed a logistic regression model with the binary dependent variable indicating if the subject’s blood pressure was uncontrolled (=0) or controlled (=1). The explanatory variable of interest for the regression models was a categorical variable indicating if the BP reading was for the initial visit (=0) or a follow-up visit (=1). The unadjusted odds of controlled blood pressure was estimated using a logistic model:

$$\frac{p_{control}}{1 - p_{control}} =exp (\beta_{0}+ \beta_{1}{followup}_{sv})$$

with p_control_ is the probability that the blood pressure is under control (SBP <=140 and DBP <= 90).

To estimate the adjusted effects, we controlled for subject fixed effects. The fixed effects would control for observed (age, sex, previous diagnosis status, pharmacy) and unobserved fixed individual characteristics. The standard errors were clustered at the pharmacy level in all models.

**Supplementary Figure 1: Geographical map of the selected blocks within Bhojpur for the study**


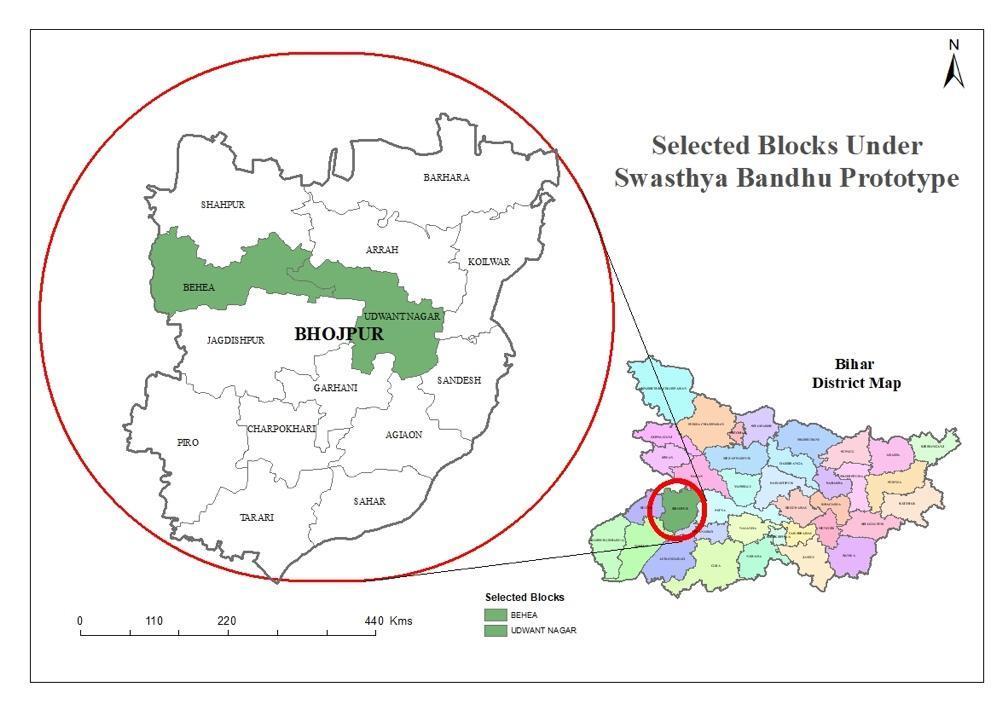


**Supplementary Table S1: Baseline characteristics of subjects with at least one follow-up visit**

| Parameter | All | Previously Undiagnosed | Previously Diagnosed |
| --- | --- | --- | --- |
| n | 129 | 37 | 92 |
| Age mean (SD) | 57.34 (12.36) | 57.27 (13.91) | 57.37 (11.69) |
| Females n (%) | 57 (44.2%) | 13 (35.1%) | 44 (47.8%) |
| Systolic BP mean (SD) | 160.46 (27.37) | 160.84 (25.41) | 160.31 (28.1) |
| Diastolic BP mean (SD) | 90.93 (14.49) | 91.92 (12.61) | 90.54 (15.15) |
| Controlled BP n (%) | 25 (19.4%) | 5 (13.5%) | 20 (21.7%) |

**Supplementary Table S2A: Comparison of baseline characteristics of Screened vs enrolled with at least one follow-up visit.**

| Parameter |  | Enrolled with at least one follow-up visit |  |
| --- | --- | --- | --- |
|  | Screened Subjects |  | p-value |
| n | 3403 | 129 | - |
| Age mean (SD) | 47.41 (16.01) | 57.34 (12.36) | 0.000*** |
| Females n (%) | 1313 (39%) | 57 (43.85%) | 0.268 |
| Systolic BP mean (SD) | 133.92 (23.37) | 160.46 (27.37) | 0.000*** |
| Diastolic BP mean (SD) | 80.9 (12.03) | 90.93 (14.49) | 0.000*** |
| Previously  undiagnosed n (%) | 675 (20%) | 37 (28.46%) | 0.019** |
| Controlled BP n (%) | 274 (8.1%) | 25 (19.4%) | 0.000*** |

** p < 0.05 *** p < 0.001

**Supplementary Table S2B: Comparison of baseline characteristics of potentially hypertensive vs enrolled with at least one follow-up visit.**

| Parameter | Potentially Hypertensive Subjects | Enrolled with at least one follow-up visit | p-value |
| --- | --- | --- | --- |
| n | 1415 | 129 | - |
| Age (SD) | 54.02 (14.46) | 57.34 (12.36) | 0.003** |
| Females (%) | 632 (45%) | 57 (43.85%) | 0.802 |
| Systolic BP (SD) | 152.15 (23.55) | 160.46 (27.37) | 0.001** |
| Diastolic BP (SD) | 88.14 (13.17) | 90.93 (14.49) | 0.037** |
| Previously  Undiagnosed (%) | 675 (48%) | 37 (28.46%) | 0.000*** |
| Controlled BP (%) | 274 (19.4%) | 25 (19. 4%) | 0.550 |

** p < 0.05 *** p < 0.001

**Supplementary Table S3: Regression results for Systolic Blood Pressure**

|  | | | | | | | |
| --- | --- | --- | --- | --- | --- | --- | --- |
|  | A: Dependent variable: | | | | | | |
|  |  | | | | | | |
|  | Systolic Blood Pressure | | | | | | |
|  | (1) | (2) | (3) | (4) | (5) | (6) | (7) |
|  | | | | | | | |
| Followup | -11.517^***^ | -11.619^***^ | -10.080^***^ | -11.527^***^ | 18.651^**^ | -9.745^***^ | 20.108^*^ |
|  | (2.611) | (2.635) | (3.535) | (2.765) | (9.107) | (3.039) | (10.790) |
|  |  |  |  |  |  |  |  |
| Age |  | 0.455^***^ | 0.455^***^ |  | 0.792^***^ |  |  |
|  |  | (0.116) | (0.116) |  | (0.181) |  |  |
|  |  |  |  |  |  |  |  |
| Female |  | 4.231^*^ | 5.298 |  | 7.096^**^ |  |  |
|  |  | (2.404) | (3.394) |  | (3.267) |  |  |
|  |  |  |  |  |  |  |  |
| Previously Undiagnosed |  | -0.935 | 1.242 |  | 1.609 |  |  |
|  |  | (5.826) | (6.365) |  | (6.422) |  |  |
|  |  |  |  |  |  |  |  |
| Followup * Age |  |  |  |  | -0.475^***^ |  | -0.494^***^ |
|  |  |  |  |  | (0.147) |  | (0.174) |
|  |  |  |  |  |  |  |  |
| Followup * Female |  |  | -1.532 |  | -4.269 | -1.230 | -4.035 |
|  |  |  | (3.975) |  | (3.701) | (5.657) | (5.180) |
|  |  |  |  |  |  |  |  |
| Followup * Previously Undiagnosed |  |  | -3.091 |  | -3.563 | -4.407 | -4.724 |
|  |  |  | (5.685) |  | (5.637) | (6.282) | (6.316) |
|  |  |  |  |  |  |  |  |
| Constant | 160.543^***^ | 132.743^***^ | 131.676^***^ | 166.895^***^ | 111.385^***^ | 165.559^***^ | 164.675^***^ |
|  | (3.173) | (7.045) | (7.019) | (2.073) | (11.561) | (2.279) | (2.180) |
|  |  |  |  |  |  |  |  |
|  | | | | | | | |
| Subject FE | No | No | No | Yes | No | Yes | Yes |
| Observations | 441 | 441 | 441 | 441 | 441 | 441 | 441 |
| R^2^ | 0.035 | 0.074 | 0.074 | 0.562 | 0.083 | 0.563 | 0.572 |
| Adjusted R^2^ | 0.033 | 0.065 | 0.061 | 0.380 | 0.068 | 0.378 | 0.388 |
| Residual Std. Error | 27.398  (df = 439) | 26.942  (df = 436) | 26.995  (df = 434) | 21.934  (df = 311) | 26.904  (df = 433) | 21.979  (df = 309) | 21.799  (df = 308) |
| F Statistic | 16.126^***^ (df = 1; 439) | 8.669^***^ (df = 4; 436) | 5.805^***^ (df = 6; 434) | 3.094^***^ (df = 129; 311) | 5.570^***^ (df = 7; 433) | 3.040^***^ (df = 131; 309) | 3.113^***^ (df = 132; 308) |
|  | | | | | | | |
| Note: | *p<0.1; **p<0.05; ***p<0.01 | | | | | | |

**Supplementary Table S4: Regression results for Diastolic Blood Pressure**

|  | | | | | | | |
| --- | --- | --- | --- | --- | --- | --- | --- |
|  | B: Dependent variable: | | | | | | |
|  |  | | | | | | |
|  | Diastolic Blood Pressure | | | | | | |
|  | (1) | (2) | (3) | (4) | (5) | (6) | (7) |
|  | | | | | | | |
| Followup | -5.022^***^ | -4.981^***^ | -5.284^***^ | -4.675^**^ | -2.112 | -4.226^***^ | -0.807 |
|  | (1.635) | (1.665) | (1.555) | (1.966) | (5.965) | (1.488) | (6.544) |
|  |  |  |  |  |  |  |  |
| Age |  | -0.132^**^ | -0.131^**^ |  | -0.094 |  |  |
|  |  | (0.063) | (0.063) |  | (0.099) |  |  |
|  |  |  |  |  |  |  |  |
| Female |  | -0.757 | -1.448 |  | -1.249 |  |  |
|  |  | (1.357) | (2.229) |  | (2.299) |  |  |
|  |  |  |  |  |  |  |  |
| Previously Undiagnosed |  | 0.896 | 1.190 |  | 1.230 |  |  |
|  |  | (2.111) | (2.851) |  | (2.874) |  |  |
|  |  |  |  |  |  |  |  |
| Followup*Age |  |  |  |  | -0.052 |  | -0.057 |
|  |  |  |  |  | (0.090) |  | (0.099) |
|  |  |  |  |  |  |  |  |
| Followup*Female |  |  | 0.985 |  | 0.683 | 0.193 | -0.128 |
|  |  |  | (2.143) |  | (2.185) | (3.268) | (3.166) |
|  |  |  |  |  |  |  |  |
| Followup * Previously Undiagnosed |  |  | -0.410 |  | -0.462 | -1.882 | -1.918 |
|  |  |  | (2.313) |  | (2.309) | (2.574) | (2.596) |
|  |  |  |  |  |  |  |  |
| Constant | 90.907^***^ | 98.559^***^ | 98.741^***^ | 93.006^***^ | 96.500^***^ | 92.670^***^ | 92.569^***^ |
|  | (1.870) | (4.096) | (4.073) | (1.475) | (6.383) | (1.116) | (1.166) |
|  |  |  |  |  |  |  |  |
|  | | | | | | | |
| Subject FE? | No | No | No | Yes | No | Yes | Yes |
| Observations | 441 | 441 | 441 | 441 | 441 | 441 | 441 |
| R^2^ | 0.025 | 0.038 | 0.038 | 0.509 | 0.038 | 0.510 | 0.511 |
| Adjusted R^2^ | 0.023 | 0.029 | 0.025 | 0.306 | 0.023 | 0.302 | 0.301 |
| Residual Std. Error | 14.287  (df = 439) | 14.244  (df = 436) | 14.274  (df = 434) | 12.041  (df = 311) | 14.288 (df = 433) | 12.071  (df = 309) | 12.085  (df = 308) |
| F Statistic | 11.278^***^ (df = 1; 439) | 4.252^***^ (df = 4; 436) | 2.844^***^ (df = 6; 434) | 2.503^***^ (df = 129; 311) | 2.458^**^ (df = 7; 433) | 2.456^***^ (df = 131; 309) | 2.434^***^ (df = 132; 308) |
|  | | | | | | | |
| Note: | *p<0.1; **p<0.05; ***p<0.01 | | | | | | |

**Supplementary Table S5: Regression results for BP Control**

|  | | | | | | | |
| --- | --- | --- | --- | --- | --- | --- | --- |
|  | Dependent variable: | | | | | | |
|  |  | | | | | | |
|  | BP Control | | | | | | |
|  | (1) | (2) | (3) | (4) | (5) | (6) | (7) |
|  | | | | | | | |
| Followup | 2.530^***^ | 2.633^***^ | 2.543^**^ | 2.285 | 7.066^***^ | 9.422^***^ | 17.915 |
|  | (0.636) | (0.673) | (1.001) | (3.090) | (2.949) | (6.694) | (38.560) |
|  |  |  |  |  |  |  |  |
| Age |  | 0.968^***^ | 0.968^***^ | 0.966^*^ |  |  |  |
|  |  | (0.009) | (0.009) | (0.019) |  |  |  |
|  |  |  |  |  |  |  |  |
| Female |  | 0.927 | 1.244 | 1.236 |  |  |  |
|  |  | (0.206) | (0.570) | (0.576) |  |  |  |
|  |  |  |  |  |  |  |  |
| Previously Undiagnosed |  | 1.041 | 0.548 | 0.545 |  |  |  |
|  |  | (0.244) | (0.303) | (0.304) |  |  |  |
|  |  |  |  |  |  |  |  |
| Followup * Female |  |  | 0.684 | 0.690 |  | 0.348 | 0.342 |
|  |  |  | (0.354) | (0.366) |  | (0.295) | (0.291) |
|  |  |  |  |  |  |  |  |
| Followup * Previously Undiagnosed |  |  | 2.234 | 2.246 |  | 1.919 | 1.852 |
|  |  |  | (1.370) | (1.385) |  | (1.721) | (1.669) |
|  |  |  |  |  |  |  |  |
| Followup*Age |  |  |  | 1.002 |  |  | 0.989 |
|  |  |  |  | (0.022) |  |  | (0.034) |
|  |  |  |  |  |  |  |  |
| Constant | 0.240^***^ | 1.492 | 1.579 | 1.716 | 0.065^**^ | 0.049^**^ | 0.049^**^ |
|  | (0.054) | (0.870) | (0.994) | (2.046) | (0.081) | (0.068) | (0.067) |
|  |  |  |  |  |  |  |  |
|  | | | | | | | |
| Subject FE? | No | No | No | No | Yes | Yes | Yes |
| Observations | 441 | 441 | 441 | 441 | 441 | 441 | 441 |
| Log Likelihood | -270.339 | -263.560 | -262.249 | -262.245 | -125.197 | -124.047 | -123.996 |
| Akaike Inf. Crit. | 544.678 | 537.120 | 538.497 | 540.490 | 510.395 | 512.093 | 513.992 |
|  | | | | | | | |
| Note: | *p<0.1; **p<0.05; ***p<0.01 | | | | | | |

**STROBE Statement**

|  | Item No. | Recommendation | Page  No. | Relevant text from manuscript |
| --- | --- | --- | --- | --- |
| **Title and abstract** | 1 | (*a*) Indicate the study’s design with a commonly used term in the title or the abstract | 1 |  |
|  |  | (*b*) Provide in the abstract an informative and balanced summary of what was done and what was found | 2 |  |
| Introduction | | | |  |
| Background/rationale | 2 | Explain the scientific background and rationale for the investigation being reported | 4 |  |
| Objectives | 3 | State specific objectives, including any prespecified hypotheses | 4 | We studied the effect of this task-sharing intervention on the care provided to hypertensive individuals and the resulting health outcomes. |
| Methods | | | |  |
| Study design | 4 | Present key elements of study design early in the paper | 5 | Study Design, Figure 1 |
| Setting | 5 | Describe the setting, locations, and relevant dates, including periods of recruitment, exposure, follow-up, and data collection | 5,6 | Study Setting, Data Collection, |
| Participants | 6 | (*a*) *Cohort study*—Give the eligibility criteria, and the sources and methods of selection of participants. Describe methods of follow-up  *Case-control study*—Give the eligibility criteria, and the sources and methods of case ascertainment and control selection. Give the rationale for the choice of cases and controls  *Cross-sectional study*—Give the eligibility criteria, and the sources and methods of selection of participants | 5 | Study Setting |
|  |  | (*b*) *Cohort study*—For matched studies, give matching criteria and number of exposed and unexposed  *Case-control study*—For matched studies, give matching criteria and the number of controls per case |  |  |
| Variables | 7 | Clearly define all outcomes, exposures, predictors, potential confounders, and effect modifiers. Give diagnostic criteria, if applicable | 9 | Analysis |
| Data sources/ measurement | 8* | For each variable of interest, give sources of data and details of methods of assessment (measurement). Describe comparability of assessment methods if there is more than one group | 8 | Data Collection |
| Bias | 9 | Describe any efforts to address potential sources of bias | 7 | Consultations: *“To ensure equitable access…”* |
| Study size | 10 | Explain how the study size was arrived at | 6  10 | Study Setting,  Screening (Results) |

Continued on next page

| Quantitative variables | 11 | Explain how quantitative variables were handled in the analyses. If applicable, describe which groupings were chosen and why |  | 9 | Analysis |
| --- | --- | --- | --- | --- | --- |
| Statistical methods | 12 | (*a*) Describe all statistical methods, including those used to control for confounding |  | 9  Supplementary Text | Analysis  Regression Models |
|  |  | (*b*) Describe any methods used to examine subgroups and interactions |  | 9  Supplementary Text | Analysis  Regression Models |
|  |  | (*c*) Explain how missing data were addressed |  | 8 | Data Collection |
|  |  | (*d*) *Cohort study*—If applicable, explain how loss to follow-up was addressed  *Case-control study*—If applicable, explain how matching of cases and controls was addressed  *Cross-sectional study*—If applicable, describe analytical methods taking account of sampling strategy |  | 7 | Enrollments and Follow-ups |
|  |  | (*e*) Describe any sensitivity analyses |  | NA |  |
| Results | | | | | |
| Participants | 13* | (a) Report numbers of individuals at each stage of study—eg numbers potentially eligible, examined for eligibility, confirmed eligible, included in the study, completing follow-up, and analysed |  | 10,11 | Results – Operational Outcomes |
|  |  | (b) Give reasons for non-participation at each stage |  | 11 | Results – Consultation, Enrollments and Follow-ups |
|  |  | (c) Consider use of a flow diagram |  |  | Figure 2 |
| Descriptive data | 14* | (a) Give characteristics of study participants (eg demographic, clinical, social) and information on exposures and potential confounders |  |  | Table 1 |
|  |  | (b) Indicate number of participants with missing data for each variable of interest |  | 12,13 | Downstream Clinical Outcomes |
|  |  | (c) *Cohort study*—Summarise follow-up time (eg, average and total amount) |  |  | 5 months |
| Outcome data | 15* | *Cohort study*—Report numbers of outcome events or summary measures over time |  | 10,11 | Results – Operational Outcomes, Figure 2 |
|  |  | *Case-control study—*Report numbers in each exposure category, or summary measures of exposure |  |  |  |
|  |  | *Cross-sectional study—*Report numbers of outcome events or summary measures |  |  |  |
| Main results | 16 | (*a*) Give unadjusted estimates and, if applicable, confounder-adjusted estimates and their precision (eg, 95% confidence interval). Make clear which confounders were adjusted for and why they were included |  | 12,13 | Downstream Clinical Outcomes |
|  |  | (*b*) Report category boundaries when continuous variables were categorized |  |  |  |
|  |  | (*c*) If relevant, consider translating estimates of relative risk into absolute risk for a meaningful time period |  |  | NA |

Continued on next page

| Other analyses | 17 | Report other analyses done—eg analyses of subgroups and interactions, and sensitivity analyses | 12,13  Supplementary Tables | Downstream Clinical Outcomes  S3,S4,S5 |
| --- | --- | --- | --- | --- |
| Discussion | | | | |
| Key results | 18 | Summarise key results with reference to study objectives | 13,14 | First paragraph of Discussion |
| Limitations | 19 | Discuss limitations of the study, taking into account sources of potential bias or imprecision. Discuss both direction and magnitude of any potential bias | 14  17 | Second paragraph of Discussion, *“A couple of likely unobserved benefits…”*  Sixth paragraph of Discussion, “*The Bihar Pharmacist Hypertension Study was limited by…”* |
| Interpretation | 20 | Give a cautious overall interpretation of results considering objectives, limitations, multiplicity of analyses, results from similar studies, and other relevant evidence | 14 | Second paragraph of Discussion |
| Generalisability | 21 | Discuss the generalisability (external validity) of the study results | 18 | Seventh paragraph of Discussion, *“The experiences and outcomes of…*” |
| Other information | |  | | |
| Funding | 22 | Give the source of funding and the role of the funders for the present study and, if applicable, for the original study on which the present article is based | 23 | Funding |

*Give information separately for cases and controls in case-control studies and, if applicable, for exposed and unexposed groups in cohort and cross-sectional studies.

**Note:** An Explanation and Elaboration article discusses each checklist item and gives methodological background and published examples of transparent reporting. The STROBE checklist is best used in conjunction with this article (freely available on the Web sites of PLoS Medicine at http://www.plosmedicine.org/, Annals of Internal Medicine at http://www.annals.org/, and Epidemiology at http://www.epidem.com/). Information on the STROBE Initiative is available at www.strobe-statement.org.
